# Reaction of 1-propanol with Ozone in Aqueous Media

**DOI:** 10.3390/ijms20174165

**Published:** 2019-08-26

**Authors:** Erika Reisz, Agnes Tekle-Röttering, Sergej Naumov, Winfried Schmidt, Torsten C. Schmidt

**Affiliations:** 1 Faculty of Industrial Chemistry and Environmental Engineering, University “Politehnica” of Timişoara Bulevardul Vasile Pârvan Nr. 6, 300223 Timişoara, Romania; 2 Westfälische Hochschule Gelsenkirchen, Neidenburger Strasse 43, 45877 Gelsenkirchen, Germany; 3 Leibniz Institut für Oberflächenmodifizierung (IOM), Permoserstrasse 15, 04318 Leipzig, Germany; 4 Universität “Duisburg-Essen, Instrumentelle Analytische Chemie und Zentrum für Wasser- und Umweltforschung, Universitätsstrasse 5, 45117 Essen, Germany

**Keywords:** ozone, 1-propanol, hydride transfer, H-abstraction, insertion, kinetic data

## Abstract

The main aim of this work is to substantiate the mechanism of 1-propanol oxidation by ozone in aqueous solution when the substrate is present in large excess. Further goals are assessment of the products, their formation yields as well as the kinetic parameters of the considered reaction. The reaction of ozone with 1-propanol in aqueous solution occurs via hydride transfer, H-abstraction and insertion. Of these three mechanisms, the largest share is for hydride transfer. This implies the extraction of an hydride ion from the activated C−H group by O_3_ according to reaction: (C_2_H_5_)(H)(HO)C−H + O_3_ → [(C_2_H_5_)(H)(HO)C^+^ + HO_3_^−^]_cage_ → (C_2_H_5_)(H)(HO)C^+^ + HO_3_^−^. The experimentally determined products and their overall formation yields with respect to ozone are: propionaldehyde—(60 ± 3)%, propionic acid—(27.4 ± 1.0)%, acetaldehyde—(4.9 ± 0.3)%, acetic acid—(0.3 ± 0.1)%, formaldehyde—(1.0 ± 0.1)%, formic acid—(4.6 ± 0.3)%, hydrogen peroxide—(11.1 ± 0.3)% and hydroxyl radical—(9.8 ± 0.3)%. The reaction of ozone with 1-propanol in aqueous media follows a second order kinetics with a reaction rate constant of (0.64 ± 0.02) M^−1^·s^−1^ at pH = 7 and 23 °C. The dependence of the second order rate constant on temperature is described by the equation: $ln\;k_{II}=\left(27.17\pm 0.38\right)–\left(8180\pm 120\right)\times T^{-1}$, which gives the activation energy, Ea = (68 ± 1) kJ mol^−1^ and pre-exponential factor, A = (6.3 ± 2.4) × 10^11^ M^−1^ s^−1^. The nature of products, their yields and the kinetic data can be used in water treatment. The fact that the hydride transfer is the main pathway in the 1-propanol/ozone system can probably be transferred on other systems in which the substrate is characterized by C−H active sites only.

## 1. Introduction

This paper is one of a series that tries to shed some light on ozone reactions with compounds characterised by C–H active sites only, compounds known as less reactive. In this context, the main aims are to elucidate the mechanism of 1-propanol reaction with ozone in aqueous media and to provide kinetic parameters that characterize this reaction (activation energy and pre-exponential factor).

To the best of our knowledge, there are only a few data in the literature on 1-propanol/ozone aqueous system and these refer mainly to the rate constants of 1-propanol reactions with oxidants such as O_3_, HO•, and O•^−^ at temperatures between 20 and 25 °C. This information is given in Table 1.

The reason why the mechanism of this reaction is of such great interest is the assumption that hydride transfer plays an important role when ozone attack occurs at a C−H bond. This pattern has already been reported for aqueous systems such as 2-propanol/ozone [5], *tert*-butanol/ozone and formate/ozone [6]. The elementary reaction that triggers this mechanism can be written as follows [5,6,7]:

R_3_C−H + O_3_ → R_3_C^+^ + HO_3_^−^ (a)

where R stands for hydrogen, organic or functional groups, identical or different.

The interpretation of experimental data for the system 2-propanol/ozone in organic media has led to the conclusion that ozone attack at C−H bond occurs via H-abstraction [8,9,10]:

R_3_C−H + O_3_ → R_3_C• + HO_3_• (b)

The mechanism triggered by reaction (b) is not ruled out in aqueous media, but it plays a secondary role at least in the previously mentioned systems.

Other two possible interactions between ozone and substrate are insertion (reaction (c)) and electron transfer (reaction (d)):

R_3_C−H + O_3_ → R_3_C−O−O−O−H (c)

R_3_C−H + O_3_ → [R_3_C−H]•^+^ + O_3_•^−^ (d)

In order to determine which of the four mentioned pathways is the most probable, Gibbs energies of the constituent reactions and overall formation yields of the products with respect to ozone were taken into consideration.

The results of this study, mainly the kinetic data and information about the nature and yields of products can also serve practical purposes, such as the treatment of wastewaters containing 1-propanol (1-propanol may be used as a solvent or as an ingredient of degreasing agents, antifreezes, disinfectants, inks, toners, dye solutions and also as a raw material for herbicides, insecticides, cosmetics and pharmaceuticals).

## 2. Results

### 2.1. Products

#### 2.1.1. Hydroxyl Radical

**Figure 1:**
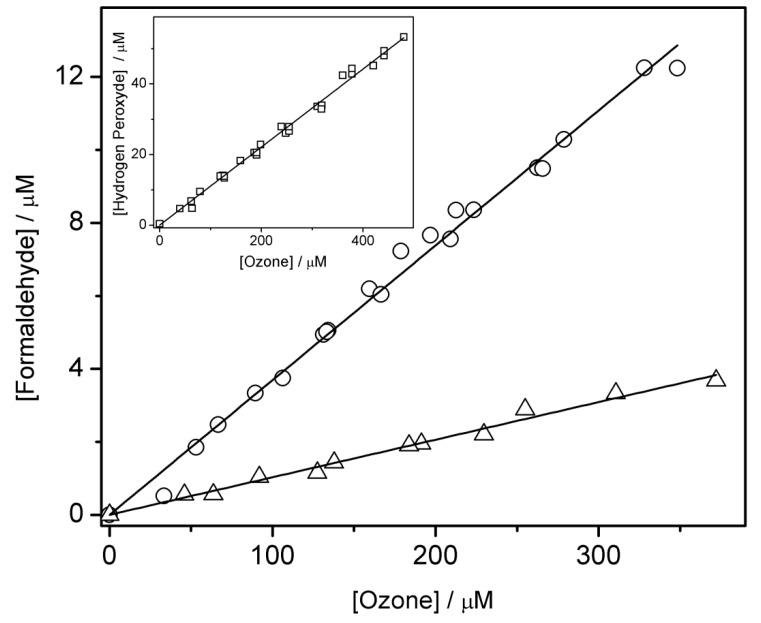
Main graph: determination of HO• formation yield with respect to ozone by using the formaldehyde formation yields in 1-propanol/ozone system in the absence (triangles) and presence of *tert*-butanol (circles) used as scavenger. Formaldehyde was determined spectrophotometrically with the Hantzsch method. [1-propanol] = 0.1 M, [*tert*-butanol] = 1 M. Inset: dependence of the formed hydrogen peroxide vs. added ozone concentrations. Hydrogen peroxide was determined spectrophotometrically by using Allen’s method. [1-propanol] = 0.1 M.

As described in Materials and Methods Section, HO• formation yield with respect to ozone was determined indirectly by assessing formaldehyde formation yields with respect to ozone in the 1-propanol/O_3_ system in the absence and presence of *tert*-butanol acting as a scavenger. In order to use this method, the reaction between substrate and O_3_ should definitely prevail over that one between *tert*-butanol and O_3_. In addition, the reaction of the substrate with HO• should be negligible with respect to that one between *tert*-butanol and HO•. Under our working conditions: [1-propanol] = 0.1 M and [*tert*-butanol] = 1 M, 98% of O_3_ reacted with 1-propanol (k(1-propanol + O_3_) = (0.64 ± 0.02) M^−1^ s^−1^ (this work) and k(*tert*-butanol + O_3_) = 1.1 × 10^−3^ M^−1^ s^−1^ [6]) and 69% of HO• was scavenged by *tert*-butanol (k(1-propanol + HO•) = 2.7 × 10^9^ M^−1^ s^−1^ [4]) and k(*tert*-butanol + HO•) = 6 × 10^8^ M^−1^ s^−1^ [11,12]). Practically, HO• formation yield with respect to ozone was calculated with the following formula:

$$\eta \left(HO\cdot \right)=2\cdot \;\frac{\left(a-0.31\cdot b\right)}{0.69}\%$$

where: the factor 2 comes from the 50% formaldehyde formation yield with respect to HO• (more details are given in the Materials and Methods section), *a* and *b* are the formaldehyde formation yields with respect to ozone in the presence and absence of *tert*-butanol, 0.69 is the part of HO• that reacts with *tert*-butanol and 0.31 the part of HO• that reacts with 1-propanol in systems that contains both substrates next to ozone. The formaldehyde formation yields with respect to ozone, *a* and *b*, are derived from the slopes of the straight lines in the graphic representation of the formed formaldehyde concentration vs. the added ozone concentration, as shown in Figure 1. Under these circumstances HO• formation yield was (9.8 ± 0.3)%.

This value indicates that the substrate is oxidized by both O_3_ (direct reaction) and HO• (indirect reaction). Due to the fact that some of the products arise on both channels and that one cannot make a clear distinction between the two contributions, the experimentally determined formation yields are called “overall formation yields”.

#### 2.1.2. Aldehydes

As part of this category, propionaldehyde was formed by oxidation, while acetaldehyde and formaldehyde resulted from oxidative cleavage of 1-propanol.

Acetaldehyde and propionaldehyde were determined by means of high performance liquid chromatography (HPLC) and formaldehyde by spectrophotometric method. Details can be found in the Materials and Methods section.

**Figure 2:**
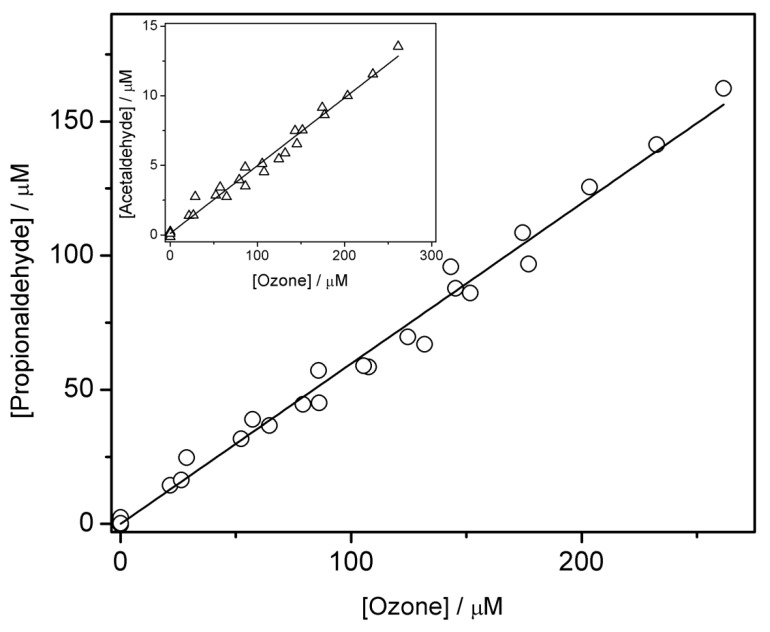
Main graph: dependence of the formed propionaldehyde versus added ozone concentrations. Inset: plot of the formed acetaldehyde versus added ozone concentrations. Propionaldehyde and acetaldehyde were determined as hydrazones by HPLC with optic detection. [1-propanol] = 0.1 M.

The formation yield of propionaldehyde was (60 ± 3)% and it was obtained from the plot of the formed propionaldehyde versus the added ozone concentrations, as shown in Figure 2.

The formation yields for formaldehyde and acetaldehyde were one order of magnitude lower than the one for propionaldehyde. Thus, the formation yield of formaldehyde was (1.0 ± 0.1)% and that one for acetaldehyde was (4.9 ± 0.3)%. These results were based on data from Figure 1 (for formaldehyde; triangles in the main graph) and the inset of Figure 2 (for acetaldehyde).

#### 2.1.3. Acids

**Figure 3:**
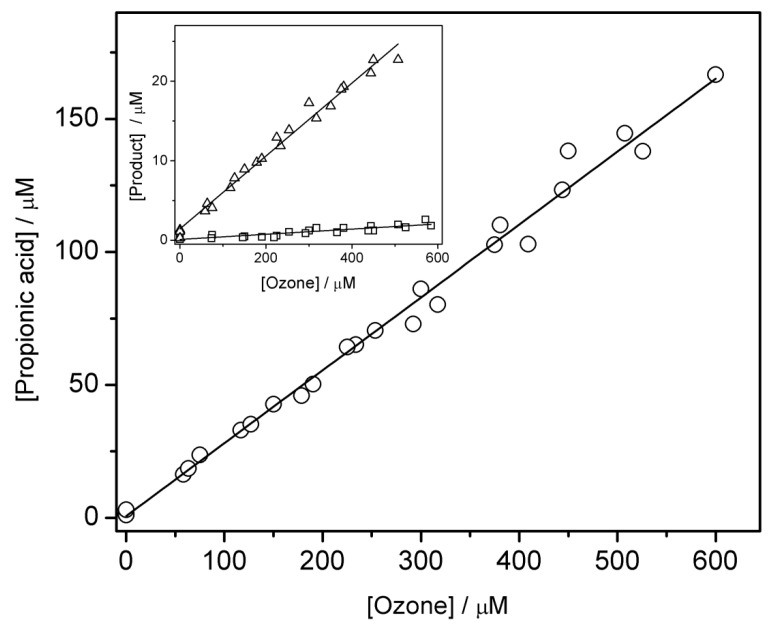
Main graph: dependence of the formed propionic acid versus added ozone concentrations. Inset: plots of the formed acetic and formic acids (squares and triangles, respectively) versus added ozone concentrations. Propionic, acetic and formic acids were determined by IC. [1-propanol] = 0.1 M.

Of this category, propionic, acetic and formic acids formed within our system. Their concentrations were determined by way of ion chromatography (IC) as described in Materials and Methods section. The formation yields with respect to ozone were as follows (27.4 ± 1.0)%—propionic acid, (0.3 ± 0.1)%—acetic acid and (4.6 ± 0.3)%—formic acid. These values were obtained based on data in Figure 3, which shows the dependence of the formed acid on opposing ozone concentrations.

#### 2.1.4. Hydrogen Peroxide

The overall H_2_O_2_ formation yield with respect to ozone was determined from the plot of the formed H_2_O_2_ concentration versus added ozone, as shown in the inset of Figure 1. The resulted value was (11.1 ± 0.3)%. The H_2_O_2_ concentration was determined spectrophotometrically, as shown in Section 4.

#### 2.1.5. Overview

Table 2 summarizes overall product formation yields in 1-propanol/O_3_ system. Firstly, these data show that the material balance is nearly closed, i.e., the sum of products formation yields with respect to O_3_ is 93%. The remaining 7% is due to the practically unmeasurable low concentration of certain products (hydroxyacetone, hydroxyacetaldehyde and 1,2-propandiol), experimental errors, values used for molar absorption coefficients, etc. Secondly, one can notice that the sum of formation yields of compounds with one carbon atom (5.6%—resulted from 1.0% for formaldehyde and 4.6% for formic acid) equals that one corresponding to compounds with two carbon atoms (5.2%—obtained from 4.9% for acetaldehyde and 0.3% for acetic acid).

### 2.2. Kinetic Data

The first part of this section refers to determining the second order rate constant of the reaction between 1-propanol and ozone at 23 °C and comparing it with corresponding data in the literature and also with values reported for other alcohols.

**Figure 4:**
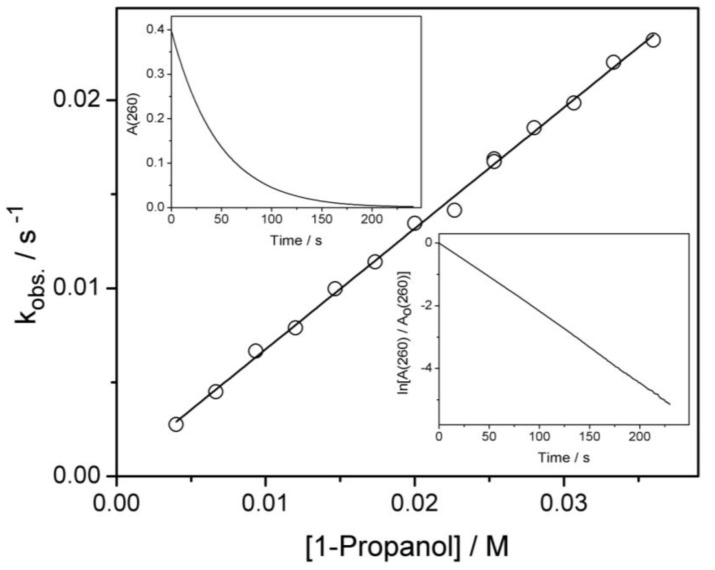
Main graph: Determination of the second order rate constant for the reaction of ozone with 1-propanol. Correlation of pseudo-first order rate constant (observed rate constant, k_obs._) with 1-propanol concentration at t = 23 °C and pH = 7 (as 1-propanol is in large excess with respect to ozone, the concentration of 1-propanol can be considered constant during reaction). Upper inset: exponential decrease of ozone concentration (measured as absorbance at 260 nm) versus time for [1-propanol] = 36 mM. Lower inset: the corresponding linear decrease of *ln* (A/A_0_) versus time.

The determination of the second order rate constant is based on graphic representation of the pseudo-first order rate constant, k_obs._, versus 1-propanol concentration, which practically remained unchanged throughout the reaction (Figure 4, main graph). To get the pseudo-first order rate constant, the variation of ozone absorbance at 260 nm was recorded as the reaction went on (Figure 4, upper inset), the natural logarithm of the ratio between current and initial absorbance was plotted versus time (Figure 4, lower inset) and finally the slope of the straight line was calculated (tg α = k_obs._).

The resulting value for the second order rate constant at pH = 7 and 23 °C is (0.64 ± 0.02) M^−1^·s^−1^.

Table 3 shows second order rate constants for reactions among O_3_ and various alcohols.

By comparing the second order rate constants as determined by Hoigné and Bader at 20 °C for primary alcohols [1], one can notice the increase of the reaction rate within series, from methanol to 1-octanol, following the increase of the electron repelling inductive effect as the number of carbon atoms within the organic chain increases. As expected, the rate constant also increases when the number of methyl groups that show electron repelling inductive effect increases. Table 3 highlights this influence for the switch from ethanol (one methyl group) to 2-propanol (two methyl groups).

**Figure 5:**
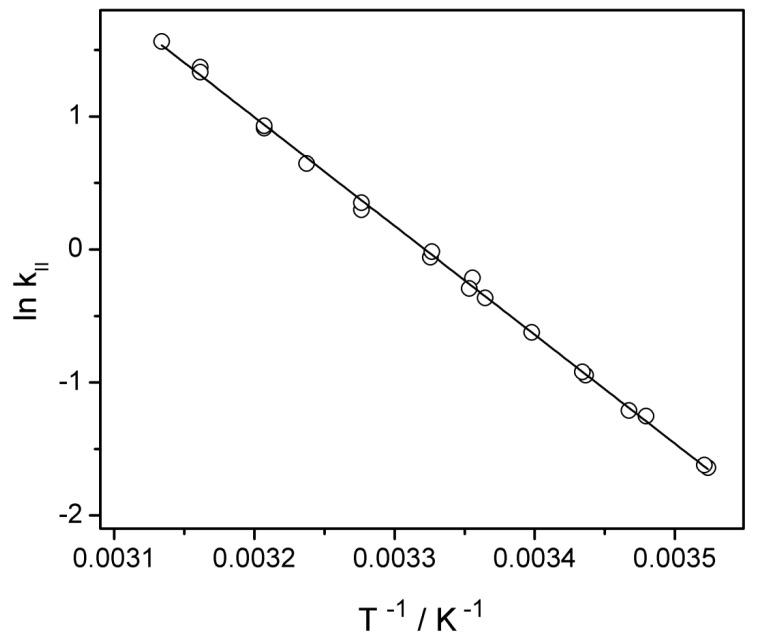
Determination of the activation energy, Ea, and pre-exponential factor, A. Dependence between the natural logarithm of the second order rate constant and inverse of absolute temperature (Arrhenius plot).

The second part of this section deals with Arrhenius graphic representation in order to derive the second order rate constants at any given temperature within the considered range and also of the activation energy and pre-exponential factor. To get this information, the second order rate constants were determined for temperatures between 10 and 45 °C and the natural logarithm of the second order rate constant was plotted against the inverse of absolute temperature, as in Figure 5. The explicit forms of dependencies *ln* k_II_ = f(T^−1^) and k = f(T) are:

$$ln\;k_{II}=\left(27.17\pm 0.38\right)–\left(8180\pm 120\right)\times T^{-1}$$

and

$$k_{II}=\left(6.3\pm 2.4\right)\cdot 10^{11}\cdot e^{-\frac{\left(68\pm 1\right)\cdot 10^{3}}{R\cdot T}}$$

These relations lead to (68 ± 1) kJ mol^−1^ activation energy and (6.3 ± 2.4) × 10^11^ M^−1^ s^−1^ pre-exponential factor.

The second order rate constant at 20 °C and pH = 7 derived from the dependence k = f(T) was (4.7 ± 0.2) × 10^−1^ M^−^^1^ s^−^^1^ and it was in agreement with that reported by Hoigné and Bader of (3.7 ± 0.4) × 10^−1^ M^−1^ s^−^^1^ at the same temperature and pH = 2 [1].

## 3. Discussion

### 3.1. Mechanism Initiated by the Direct Reaction of O_3_ with 1-propanol

#### 3.1.1. Hydride Transfer

In the first step, O_3_ extracts a hydride ion from the activated C−H group thereby forming a carbocation and a hydrogen trioxide anion, HO_3_^−^; this ion couple is kept together for some time within the solvent cage (reaction (1)). Further, these ions can recombine in the solvent cage forming α-hydroxyalkylhydrotrioxide (reaction (2), overall Gibbs energy for reactions (1) and (2), ΔG = −192 kJ mol^−1^), or they can escape from the cage (reaction (3), overall Gibbs energy for reactions (1) and (3), ΔG = −167 kJ mol^−1^).

(C_2_H_5_)(H)(HO)C−H + O_3_ → [(C_2_H_5_)(H)(HO)C^+^ + HO_3_^−^]_cage_ (1)

[(C_2_H_5_)(H)(HO)C^+^ + ^−^O_3_H]_cage_ → (C_2_H_5_)(H)(HO)C−O−O−OH (2)

[(C_2_H_5_)(H)(HO)C^+^ + HO_3_^−^]_cage_ → (C_2_H_5_)(H)(HO)C^+^ + HO_3_^−^ (3)

One can notice that both successions of reactions ((1) plus (2) and (1) plus (3)) are highly exergonic and thus thermodynamically favoured. The fate of trioxide will be discussed as part of the paragraph that deals with the insertion mechanism.

The carbocation resulted following reaction (3) might rearrange by forming protonated propionaldehyde (reaction (4), ΔG = 0 kJ mol^−1^), a species that is likely to lead to propionaldehyde by deprotonation (reaction (5), ΔG = −40 kJ mol^−1^).

(C_2_H_5_)(H)(HO)C^+^ → (C_2_H_5_)(H)C=OH^+^ (4)

(C_2_H_5_)(H)C=OH^+^ → (C_2_H_5_)(H)C=O + H^+^ (5)

At the circumneutral pH of our experiments, hydrogen trioxide anion that resulted in reaction (3) will protonate forming hydrogen trioxide (reaction (6), pKa(H_2_O_3_) = (9.5 ± 0.2) at 20 °C, k_−_ = 9.2 s^−1^, k_+_ = 10^10^ M^−1^ s^−1^ [13,14,15]), an unstable species in water, which decays by acid catalysis (reaction (7), k = 6 M^−1^ s^−1^ [13,14,15], ΔG = −234 kJ mol^−1^). The hydrogen trioxide anion itself undergoes decay to singlet oxygen and hydroxide ion (reaction (8), ΔG = −40 kJ mol^−1^) [13].

HO_3_^−^ + H^+^ ⇄ H_2_O_3_ (6)

H_2_O_3_ + H^+^ → H_3_O^+^ + O_2_ (7)

HO_3_^−^ → HO^−^ + ^1^O_2_ (8)

Given the reaction sequence initiated by a hydride ion transfer from 1-propanol to ozone (reactions (1), (3)–(8)), it follows that the sole stable product is propionaldehyde (except for oxygen). As the overall formation yield for propionaldehyde is (60 ± 3)%, it means that maximum (60 ± 3)% of ozone has reacted with 1-propanol according to this mechanism (practically, by hydride transfer followed by ions escaping from the solvent cage).

#### 3.1.2. Insertion

This mechanism involves the intercalation of one O_3_ molecule between carbon and hydrogen atoms of an activated C−H group, when α-hydroxyalkylhydrotrioxide forms (reaction (9), ΔG = −192 kJ mol^−1^). This trioxide might also form within the solvent cage via a hydride transfer mechanism (by recombination of the previously formed ion pair) and/or via H-abstraction (by recombination of the radical pair).

(C_2_H_5_)(H)(HO)C−H + O_3_ → (C_2_H_5_)(H)(HO)C−O−O−OH (9)

The resulted α-hydroxyalkylhydrotrioxide is likely to cleave to a secondary α-hydroxyalkyloxyl radical and a hydroperoxyl radical (reaction (10), ΔG = −14 kJ mol^−1^) or to a secondary α-hydroxyalkylperoxyl radical and a hydroxyl radical (reaction (11), ΔG = 46 kJ mol^−1^). Both Gibbs energies of reactions and lengths of O−O bonds (1.442 Å for O−OOH and 1.426 Å for OO−OH) indicate that reaction (10) is favored over reaction (11).

(C_2_H_5_)(H)(HO)C−O−O−OH → (C_2_H_5_)(H)(HO)C−O• + HO_2_• (10)

(C_2_H_5_)(H)(HO)C−O−O−OH → (C_2_H_5_)(H)(HO)C−O−O• + HO• (11)

The α-hydroxyalkyloxyl radical might undergo 1,2-H shift (reaction (12), ΔG = −30 kJ mol^−1^) or β-fragmentation (reaction (13), ΔG = −82 kJ mol^−1^)

(C_2_H_5_)(H)(HO)C−O• → (C_2_H_5_)(HO)_2_C• (12)

(C_2_H_5_)(H)(HO)C−O• → •C_2_H_5_ + HCOOH (13)

When O_2_ is present, as in our system, it reacts with the dihydroxyalkyl radical formed in reaction (12), leading to a α-hydroxyalkylperoxyl radical (reaction (14), ΔG = −101 kJ mol^−1^), which itself is in equilibrium with the corresponding radical anion (reaction (15)).

(C_2_H_5_)(HO)_2_C• + O_2_ → (C_2_H_5_)(HO)_2_C−O−O• (14)

(C_2_H_5_)(HO)_2_C−O−O• + HO^−^ ⇄ (C_2_H_5_)(HO)(O^−^)C−O−O• + H_2_O (15)

The literature shows that as a general rule, α-hydroxyalkylperoxyl radicals, R_2_(HO)C−O−O•, eliminate HO_2_• following a first order reaction that may be slow, the reaction rate constant depending on the nature of substituents [16,17,18]. The corresponding radical anions, R_2_(O^–^)C−O−O•, eliminate O_2_•^−^ very fast, following a second order process. Actually, this process consists of the equilibrium between the protonated and deprotonated species and O_2_•^−^ elimination from deprotonated species, where the first step is the rate limiting one [7,19]. More details about HO_2_•/O_2_•^−^ elimination are provided in Supplementary Materials.

Thus, the α-hydroxyalkylperoxyl radical formed in reaction (14) is likely to eliminate HO_2_• according to reaction (16) (ΔG = −98 kJ mol^−1^) and the corresponding α-hydroxyalkylperoxyl radical anion might eliminate O_2_•^−^ as shown in reaction (17) (ΔG = −11 kJ mol^−1^). Both reactions lead to propionic acid as a product.

(C_2_H_5_)(HO)_2_C−O−O• → (C_2_H_5_)COOH + HO_2_• (16)

(C_2_H_5_)(HO)(O^−^)C−O−O• → (C_2_H_5_)COOH + O_2_•^−^ (17)

Between HO_2_• and O_2_•^−^, as formed in reactions (16) and (17), one can write equilibrium (18) characterized by pKa = (4.8 ± 0.1), which indicates that in our system (pH ~ 7), O_2_•^−^ is the predominant species [20]. This could react with O_3_ in excess, according to reaction (19), thus forming O_3_•^−^, which is a precursor of HO•. Given that in our experiments the substrate is in large excess ([2-propanol] = 10^−1^ M, [O_3_] ≅ 5 × 10^−4^ M), reaction (19) is less important, which means that insertion mechanism practically does not lead to the formation of HO•.

HO_2_• ⇄ O_2_•^−^ + H^+^ (18)

O_2_•^−^ + O_3_ → O_2_ + O_3_•^−^ (19)

HO_2_•/O_2_•^−^ are unstable species that can undergo bimolecular decay according to reactions (20) (k = (8.3 ± 0.7) × 10^5^ M^−1^ s^−1^) and (21) (k = (9.7 ± 0.6) × 10^7^ M^−1^ s^−1^) thereby forming H_2_O_2_/HO_2_^−^ [20]. However, a bimolecular decay of O_2_•^−^ does not take place to any significant extent [7].

2 HO_2_• → H_2_O_2_ + O_2_ (20)

HO_2_• + O_2_•^−^ → HO_2_^−^ + O_2_ (21)

According to reactions (9)–(21), it follows that the products resulted by the insertion mechanism are: propionic acid, formic acid, hydrogen peroxide, acetaldehyde and acetic acid (the last two compounds result following the oxidation of ethyl radical as a product of reaction (13)). The overall formation yields of these products are: (27.4 ± 1.0)%—propionic acid, (4.6 ± 0.3)%—formic acid, (11.1 ± 0.3)%—hydrogen peroxide, (4.9 ± 0.3)%—acetaldehyde and (0.3 ± 0.1)%—acetic acid. From these formation yields it follows that trioxide is formed. However, this finding does not clarify whether trioxide forms following the insertion mechanism or by recombination of the ions formed by hydride transfer or of the radicals resulted from H-abstraction.

#### 3.1.3. H-abstraction

This mechanism involves taking away one hydrogen atom from the activated C–H group as shown in reaction (22), when α-hydroxyalkyl and hydrogen trioxide radicals are likely formed. The radical pair is kept together for some time by the solvent cage where they can recombine to form trioxide (reaction (23), overall Gibbs energy for reactions (22) and (23), ΔG = −192 kJ mol^−1^), or they can escape (reaction (24), overall Gibbs energy for reactions (22) and (24), ΔG = −19 kJ mol^−1^). One can notice that both pathways are exergonic and thus thermodynamically possible; the formation of trioxide is thermodynamically favored given the one order of magnitude higher Gibbs energy (absolute value) than that for the formation of free radicals.

(C_2_H_5_)(H)(HO)C−H + O_3_ → [(C_2_H_5_)(H)(HO)C• + HO_3_•]_cage_ (22)

[(C_2_H_5_)(H)(HO)C• + •O_3_H]_cage_ → (C_2_H_5_)(H)(HO)C−O−O−OH (23)

[(C_2_H_5_)(H)(HO)C• + HO_3_•]_cage_ → (C_2_H_5_)(H)(HO)C• + HO_3_• (24)

The fate of α-hydroxyalkyltrioxide has already been presented in the paragraph that deals with insertion mechanism (reactions (10)–(17)).

The α-hydroxyalkyl radical formed in reaction (24) might further reacts with oxygen that is in high concentration in our system, thereby most likely leading to α-hydroxyalkylperoxyl radical (reaction (25), ΔG = −95 kJ mol^−1^). This species is in equilibrium with the corresponding radical anion (reaction (26)). Both the protonated and deprotonated species lead to propionaldehyde by elimination of HO_2_• and O_2_•^−^, respectively (reaction (27) characterized by ΔG = −27 kJ mol^−1^ and reaction (28) with ΔG = −31 kJ mol^−1^).

(C_2_H_5_)(H)(HO)C• + O_2_ → (C_2_H_5_)(H)(HO)C−O−O• (25)

(C_2_H_5_)(H)(HO)C−O−O• + HO^−^ ⇄ (C_2_H_5_)(H)(O^−^)C−O−O• + H_2_O (26)

(C_2_H_5_)(H)(HO)C−O−O• → (C_2_H_5_)(H)C=O + HO_2_• (27)

(C_2_H_5_)(H)(O^−^)C−O−O• → (C_2_H_5_)(H)C=O + O_2_•^−^ (28)

As has already been shown at the insertion mechanism:

- There is an acid-base equilibrium between HO_2_• and O_2_•^−^ (reaction (18));
- At the circumneutral pH within our system, O_2_•^−^ is the predominant species (pKa(HO_2_•) = (4.8 ± 0.1));
- Reaction between two HO_2_• moles or between one HO_2_• and one O_2_•^−^ moles leads to the formation of H_2_O_2_ and HO_2_^−^, respectively (reactions (20) and (21));
- Given the large excess of substrate up against ozone within our system, reaction between O_2_•^−^ and O_3_ is not important and it follows that O_3_•^−^ (as a precursor of HO•) does not form practically via this pathway.

Due to the fact that α-hydroxyalkylperoxyl radical, formed in reaction (25), is a secondary peroxyl radical, it might also dimerize (reaction (29), ΔG = 94 kJ mol^−1^):

2 (C_2_H_5_)(H)(HO)C−O−O• ⇄ (C_2_H_5_)(H)(HO)C−O−O−O−O−C(OH)(H)(C_2_H_5_) (29)

The formed tetroxide is actually a short lived intermediate that further might:

- Reform the α-hydroxyalkylperoxyl radical (reverse of reaction (29), ΔG = −94 kJ mol^−1^);
- Eliminate O_2_, thus forming two α-hydroxyalkyloxyl radicals (reaction (30), ΔG = −180 kJ mol^−1^) whose fate is described by the succession of reactions (12)–(17) at insertion mechanism;
  (C_2_H_5_)(H)(HO)C−O−O−O−O−C(OH)(H)(C_2_H_5_) → 2 (C_2_H_5_)(H)(HO)C−O• + O_2_ (30)
- Decay via Russel reaction that involves a transition state with a six-membered ring (reaction (31), ΔG = −583 kJ mol^−1^) [7,21]. Given that the diol formed in reaction (31) eliminates one water molecule intramolecularly according to reaction (32) (ΔG = −52 kJ mol^−1^), the products are propionaldehyde, propionic acid, and oxygen in a 1:1:1 ratio;

(C_2_H_5_)(H)(HO)C−O−O−O−O−C(OH)(H)(C_2_H_5_) → (C_2_H_5_)(H)(HO)_2_C + (C_2_H_5_)COOH + O_2_ (31)

(C_2_H_5_)(H)(HO)_2_C → (C_2_H_5_)(H)C=O + H_2_O (32)

- Decay via Bennett reaction that involves a transition state with two five-membered rings (reaction (33), ΔG = −752 kJ mol^−1^) [7,17,22]. The products that form via this reaction are propionic acid and hydrogen peroxide in a ratio of 2:1.

(C_2_H_5_)(H)(HO)C−O−O−O−O−C(OH)(H)(C_2_H_5_) → 2 (C_2_H_5_)COOH + H_2_O_2_ (33)

The Supplementary Materials describes α-hydroxyalkylperoxyl radicals’ transformation to products, according to Russel and Bennett mechanisms and the corresponding energy diagram. As already suggested, these processes occur in accordance with the following succession: Reactants → First Transition State → Intermediate (Tetroxide) → Second Transition State → Products.

Between HO_3_• formed in reaction (24) and the corresponding anion, O_3_•^−^, one can write the equilibrium (34). Given the pKa = −2 of this reaction, it follows that HO_3_• is a very strong acid and O_3_•^−^ is the predominant species in our system [7,15,23,24,25,26]. The deprotonation occurs in the timescale of 10^−12^ s (reaction (34), k_+_ = 10^12^ s^−1^) [23,24]. Under the same timescale, i.e., in competition with deprotonation, HO_3_• decays to HO• and O_2_ (reaction (35)) [7].

HO_3_• ⇄ O_3_•^−^ + H^+^ (34)

HO_3_• → HO• + O_2_ (35)

O_3_•^−^ formed by deprotonation also acts as HO• precursor, but it decays in a larger timescale [6,24,25]. In the first step, O_3_•^−^ cleaves to O•^−^ and O_2_ (reaction (36), characterized by K = 5.5 × 10^−7^ M, k_+_ = (1.94 ± 0.16) × 10^3^ s^−1^, k_−_ = 3.5 × 10^9^ M^−1^ s^−1^) [7,24,25,27] and then O•^−^ is protonated (reaction (37), pKa = (11.84 ± 0.08)) [27].

O_3_•^−^ ⇄ O_2_ + O•^−^ (36)

O•^−^ + H^+^ ⇄ HO• (37)

Hydroxyl radicals could undergo bimolecular decay thus leading to hydrogen peroxide (reaction (38), k = 7.7 × 10^9^ M^−1^ s^−1^ [28]). Due to the fact that the substrate is in very large concentration in our system ([1-propanol] = 0.1 M) it can be expected that HO• reacts mainly with the substrate and that reaction (38) is negligibly. The interaction of hydroxyl radical with the substrate is discussed at the paragraph no. 3.2. Mechanism initiated by the reaction of HO• with 1-propanol. It is also shown in Figure 7.

2 HO• → H_2_O_2_ (38)

The products according to this mechanism are as follows: propionaldehyde, propionic acid, acetaldehyde, acetic acid, formic acid, hydrogen peroxide and hydroxyl radical. The overall formation yields, as experimentally determined, are as follows: propionaldehyde—(60 ± 3)%, propionic acid—(27.4 ± 1.0)%, acetaldehyde—(4.9 ± 0.3)%, acetic acid—(0.3 ± 0.1)%, formic acid—(4.6 ± 0.3)%, hydrogen peroxide—(11.1 ± 0.3)% and hydroxyl radical—(9.8 ± 0.3)%.

Due to the fact that HO• practically forms only via this mechanism, ozone consumption yield according to H-abstraction is expected to be equal to HO• formation yield, about 10%.

#### 3.1.4. Electron Transfer

Ozone might also react with 1-propanol by withdrawing one electron when a radical cation and an ozonide anion are likely formed (reaction (39), ΔG = 100 kJ mol^−1^).

(C_2_H_5_)H_2_C−OH + O_3_ → [(C_2_H_5_)H_2_C−OH]•^+^ + O_3_•^−^ (39)

The radical cation might lose one proton (reaction (40), ΔG = −41 kJ mol^−1^) and the resulting alkyloxyl radical is likely to undergo β-fragmentation (reaction (41), ΔG = −19 kJ mol^−1^) and 1,2-H shift (reaction (42), ΔG = −48 kJ mol^−1^).

[(C_2_H_5_)H_2_C−OH]•^+^ → (C_2_H_5_)H_2_C−O• + H^+^ (40)

(C_2_H_5_)H_2_C−O• → •C_2_H_5_ + H_2_C=O (41)

(C_2_H_5_)H_2_C−O• → (C_2_H_5_)(H)(HO)C• (42)

The hydroxyalkyl radical resulted following 1,2-H shift (reaction (42)), would further react according to the sequence formed from reactions (25)–(33).

The ozonide anion formed in reaction (39) would lead to HO• according to reactions (34)–(37).

Practically, the mechanism triggered by the electron transfer is ruled out, as suggested by the fact that the first step is strongly endergonic (ΔG = 100 kJ mol^−1^). One can notice that all products that could form via electron transfer also form through other mechanisms.

#### 3.1.5. Overview

**Figure 6:**
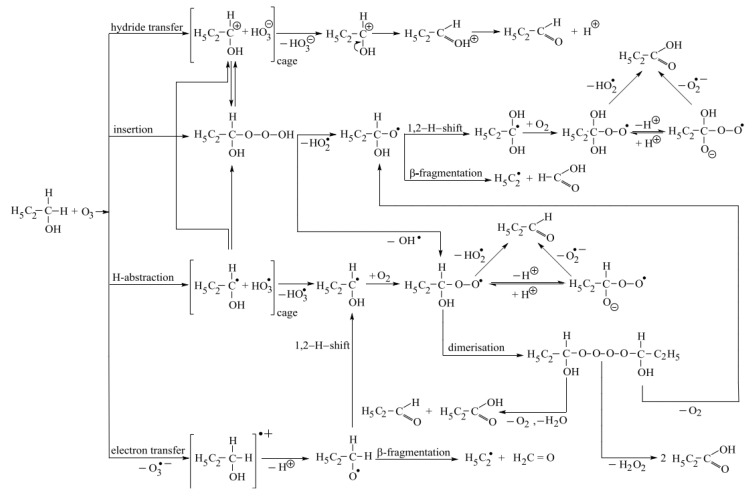
Pathways triggered by the direct reactions of ozone with 1-propanol.

The possible pathways initiated by the direct reactions of ozone with 1-propanol are shown in Figure 6.

Based on the above-mentioned discussion, it follows that of the four pathways by which ozone can react with 1-propanol directly, hydride transfer, insertion and H-abstraction are confirmed both by the thermodynamic data and quite high formation yields of the expected products. In the case of electron transfer, the highly positive value of Gibbs energy (ΔG = 100 kJ mol^−1^) suggests that this reaction does not occur, which is also confirmed by the low overall formation yield of HO•, which actually forms via H-abstraction (for the first step, when HO_3_• (HO• precursor) forms, Gibbs energy is ΔG = −19 kJ mol^−1^).

By H-abstraction, 10% of the available ozone react and this percentage is given by HO• formation yield. The stable products as part of this pathway are propionaldehyde, propionic acid, acetaldehyde, acetic acid, formic acid and hydrogen peroxide. One can notice that although the overall formation yield with respect to ozone for propionic acid and propionaldehyde are (27.4 ± 1.0)% and (60 ± 3)%, respectively, maximum 10% of the available ozone leads to the formation of these products by H-abstraction.

Hydride transfer mechanism leads only to propionaldehyde and due to the very high overall formation yield of this species, (60 ± 3)%, it means that, although it might also form by other pathways (H-abstraction and HO• attack at 1-propanol), hydride transfer is the main mechanism to a maximum 60% share of the studied mechanisms.

Insertion mechanism is likely to lead to propionic acid, acetic acid, acetaldehyde, formic acid and hydrogen peroxide. Given that the overall formation yield of propionic acid with respect to ozone is (27.4 ± 1.0)%, it follows that insertion is an important pathway, although this product can form also through H-abstraction and HO• attack at 1-propanol.

One must note that the three thermodynamically possible pathways are interconnected. Thus, the radical pair resulting from H-abstraction might undergo electron transfer, leading to the ion-pair resulting from hydride transfer (ΔG = −148 kJ mol^−1^). Also the radical pair might recombine to form the trioxide (ΔG = −173 kJ mol^−1^). In addition, an equilibrium is likely to be possible between the caged ion pair formed by hydride transfer and the trioxide formed after insertion. The reason for this is that the solvation energies of the ions might be underestimated by more than the difference between the Gibbs energies of the two reactions (ΔG = −167 kJ mol^−1^ for hydride transfer and ΔG = −192 kJ mol^−1^ for insertion).

#### 3.1.6. Comparison between 1-propanol/ozone and 2-propanol/ozone systems

The most important similarities or dissimilarities between the two aqueous systems are as follows:

- Carbonyl compounds with three carbon atoms formation yields with respect to ozone was very high for 2-propanol/ozone system (acetone formation yield was 87%) and decreased by 30% for the 1-propanol/ozone system (propionaldehyde formation yield was 60%). In the case of the 1-propanol/ozone system, propionic acid was also formed with a yield of 27%.
- Hydride transfer played a very important role for both systems, however the share of this mechanism was higher for the 2-propanol/ozone system (around 90% as opposed to maximum 60% for 1-propanol/ozone). The overall share of H-abstraction and insertion for 1-propanol/ozone system was higher or equal to 27% (propionic formation yield) and only a few percent for 2-propanol/ozone system.
- The second order rate constant of the reaction between 2-propanol and O_3_ at 23 °C, k_II_ = (2.7 ± 0.1) M^−1^ s^−1^, was roughly four times higher than that between 1-propanol and O_3_, k_II_ = (6.4 ± 0.2) × 10^−1^ M^−1^ s^−1^.
- Hydroxyl radicals formation yield was about four times higher for 1-propanol/ozone system, (9.8 ± 0.3)%, than that for 2-propanol/ozone, (2.4 ± 0.5)%. This information showed that the H-abstraction share was roughly four times higher in 1-propanol/ozone system than in 2-propanol/ozone (H-abstraction share was equal to HO• formation yield) and highlighted the increase of the importance of reactions between HO• and substrate for 1-propanol/ozone as against 2-propanol/ozone.

### 3.2. Mechanism Initiated by the Reaction of HO• with 1-propanol

Asmus and co-workers have found as part of their pulse radiolysis studies, that HO• attack on 1-propanol occurs at C(α)—53.4%, C(β)—46% and OH—less than 0.5% [29]. The corresponding reactions are (43), (44) and (45), characterized by the following Gibbs energies: −123 kJ mol^−1^, −107 kJ mol^−1^ and −76 kJ mol^−1^, respectively. The attack of HO• on hydroxyl group will be neglected in the following discussion due to the low occurrence yield.

(C_2_H_5_)(H)(HO)C−H + HO• → (C_2_H_5_)(H)(HO)C• + H_2_O (43)

((HO)CH_2_)(CH_3_)(H)C−H + HO• → ((HO)CH_2_)(CH_3_)(H)C• + H_2_O (44)

(C_2_H_5_)H_2_C−O−H + HO• → (C_2_H_5_)H_2_C−O• + H_2_O (45)

One can notice that the radical resulted in reaction (43) is the same as the one formed after H-abstraction (reactions (22) and (24)) and its fate can be followed in the paragraphs that deals with H-abstraction mechanism (reactions (25)–(33)) and insertion mechanism (reactions (12)–(17)). The resulting products (from the reaction of 1-propanol with both O_3_ and HO•) and the corresponding overall formation yields are: propionaldehyde—(60 ± 3)%, propionic acid—(27.4 ± 1.0)%, acetaldehyde—(4.9 ± 0.3)%, acetic acid—(0.3 ± 0.1)%, formic acid—(4.6 ± 0.3)% and hydrogen peroxide—(11.1 ± 0.3)%.

The radical formed in reaction (44) might add O_2_, which is in high concentration in our system, thereby leading to a peroxyl radical (reaction (46), ΔG = −100 kJ mol^−1^).

((HO)CH_2_)(CH_3_)(H)C• + O_2_ → ((HO)CH_2_)(CH_3_)(H)C−O−O• (46)

Given that this peroxyl radical does not contain the hydroxyl group in α-position, it cannot eliminate HO_2_•/O_2_•^−^ but, as a secondary peroxyl radical, it might dimerise (reaction (47), ΔG = 85 kJ mol^−1^).

2 ((HO)CH_2_)(CH_3_)(H)C−O−O• ⇄ ((HO)CH_2_)(CH_3_)(H)C−O−O−O−O−C(H)(CH_3_)(CH_2_(OH)) (47)

The formed tetroxide is a short-lived intermediate that might: (a) reform the peroxyl radical (reverse of reaction (47), ΔG = −85 kJ mol^−1^), (b) eliminate oxygen (reaction (48), ΔG = −134 kJ mol^−1^ and the Russel reaction (49), ΔG = −505 kJ mol^−1^) and (c) eliminate H_2_O_2_ (Bennett reaction (50), ΔG = −627 kJ mol^−1^).

((HO)CH_2_)(CH_3_)(H)C−O−O−O−O−C(H)(CH_3_)(CH_2_(OH)) → 2 ((HO)CH_2_)(CH_3_)(H)C−O• + O (48)

((HO)CH_2_)(CH_3_)(H)C−O−O−O−O−C(H)(CH_3_)(CH_2_(OH)) → ((HO)CH_2_)(CH_3_)(H)C−OH + ((HO)CH_2_)(CH_3_)C=O + O_2_ (49)

((HO)CH_2_)(CH_3_)(H)C−O−O−O−O−C(H)(CH_3_)(CH_2_(OH)) → 2 ((HO)CH_2_)(CH_3_)C=O + H_2_O_2_ (50)

The oxyl radical which occurred in reaction (48) is likely to decay by β-fragmentation (reaction (51), ΔG = −21 kJ mol^−1^ and reaction (52), ΔG = −60 kJ mol^−1^) and it might undergo 1,2-H shift (reaction (53), ΔG = −48 kJ mol^−1^).

((HO)CH_2_)(CH_3_)(H)C−O• → ((HO)CH_2_)(H)C=O + •CH_3_ (51)

((HO)CH_2_)(CH_3_)(H)C−O• → (HO)H_2_C• + (CH_3_)(H)C=O (52)

((HO)CH_2_)(CH_3_)(H)C−O• → ((HO)CH_2_)(CH_3_)(HO)C• (53)

Reaction (53) is followed by an O_2_ addition to the alkyl radical, when α-hydroxyalkylperoxyl radical forms (reaction (54), ΔG = −69 kJ mol^−1^). This is in equilibrium with the corresponding radical anion, according to reaction (55). Further, both protonated and deprotonated species form hydroxyacetone by eliminating HO_2_•/O_2_•^−^ (reaction (56) characterised by ΔG = −46 kJ mol^−1^ and reaction (57) with ΔG = −45 kJ mol^−1^).

((HO)CH_2_)(CH_3_)(HO)C• + O_2_ → ((HO)CH_2_)(CH_3_)(HO)C−O−O• (54)

((HO)CH_2_)(CH_3_)(HO)C−O−O• + HO^−^ ⇄ ((HO)CH_2_)(CH_3_)(^−^O)C−O−O• + H_2_O (55)

((HO)CH_2_)(CH_3_)(HO)C−O−O• → ((HO)CH_2_)(CH_3_)C=O + HO_2_• (56)

((HO)CH_2_)(CH_3_)(^−^O)C−O−O• → ((HO)CH_2_)(CH_3_)C=O + O_2_•^−^ (57)

The products that might form via HO• attack on 1-propanol in β position are: 1,2-propandiol, hydroxyacetone, acetaldehyde, hydroxyacetaldehyde, formaldehyde, formic acid and hydrogen peroxide. Out of these products, only the overall formation yields with respect to ozone of a few were determined, as follows: acetaldehyde—(4.9 ± 0.3)%, formaldehyde—(1.0 ± 0.1)%, formic acid—(4.6 ± 0.3)% and hydrogen peroxide—(11.1 ± 0.3)%.

Due to the relatively low HO• formation yield with respect to ozone, (9.8 ± 0.3)%, and taking into account that the yields of HO• attack at C(α) and C(β) are 53.4% and 46%, respectively, it results that the products formation yields with respect to ozone might be a few percent at most for this pathway.

**Figure 7:**
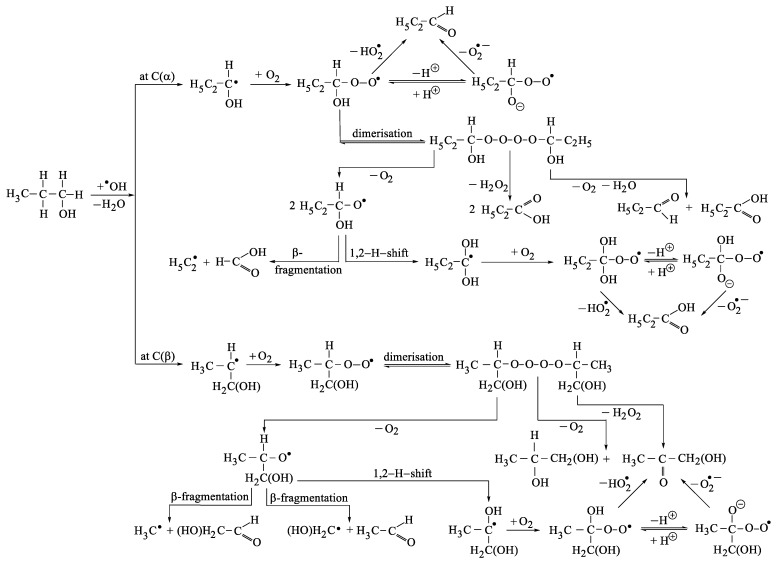
Pathways triggered by the attack of HO• on 1-propanol.

Figure 7 shows the succession of reactions triggered by the attack of HO• on 1-propanol.

## 4. Materials and Methods

All chemicals were p.a. grade from: Merck (Merck Millipore, Darmstadt, Germany), Fluka (Fluka Chemie GmbH, Buchs, Switzerland), Fischer (Fischer Scientific GmbH, Schwerte, Germany), Alfa Aesar (Alfa Aesar, Karlsruhe, Germany) or Sigma Aldrich (Sigma Aldrich Chemie GmbH, München, Germany) and they were used without further purification. More information is given in Supplementary Materials.

Ozone solutions were prepared by bubbling ozone containing oxygen stream in type I Millipore water. Ozone containing gas was obtained by means of a Sander 300.5 ozone generator (Erwin Sander Elektroapparatebau GmbH, Uetze-Eltze, Germany) fed with oxygen 6.0 from Linde (Linde A.G., München, Germany). The type I water came from a Milli Q device (Merck Millipore, Darmstadt, Germany). Ozone and 1-propanol solutions were mixed in various proportions. The concentration of ozone in both freshly prepared ozone solutions and in those that resulted after mixing with 1-propanol was determined spectrophotometrically at 260 nm by using ε(260) = 3200 M^−1^ cm^−1^ [7]. All spectrophotometric measurements were carried out with a Shimadzu UV 1800 device (Shimadzu Europa GmbH, Duisburg, Germany).

Kinetic studies were carried out in the presence of large 1-propanol excess with respect to ozone to secure half-life times of minutes. Under these circumstances, the ozone decay via HO• was negligible. More details are provided in Supplementary Materials. Practically, 1 mL ozone solution (about 15 °C) was added to 14 mL 1-propanol solution, previously brought to the required temperature, in a 5 cm thermostated quartz cuvette. The temperature of the solution was measured with a dipped thermocouple before ozone addition and after completion of the reaction. The difference between the two temperatures did not exceed 0.4 °C. Ozone decay was followed spectrophotometrically by using the time-drive mode.

The formed acids (formic, acetic and propionic acids) were quantified by means of IC with conductometric detection. The analyses were carried out by using a Metrohm 883 Basic IC plus device (Metrohm, Herisau, Switzerland) equipped with a Metrosep Organic Acids 250/7.8 column (Metrohm, Herisau, Switzerland). The eluent was 0.5 mM sulfuric acid and its flow rate, 0.5 mL min^−1^. Under these conditions, the retention times were: 13.0 (formic acid), 15.2 (acetic acid) and 17.8 (propionic acid) minutes. The suppressor was regenerated with 30 mM lithium chloride solution.

The concentration of the formed aldehydes (formaldehyde, acetaldehyde and propionaldehyde) was determined by HPLC-DAD. To that end, derivatization of the aldehydes with 2,4-dinitrophenylhydrazine (2,4-DNPH) to 2,4-dinitrophenylhydrazones was used [30]. Practically, a solution resulted by adding 0.2 mL 2,4-dinitrophenylhydrazine in acetonitrile (6 mM) and 0.1 mL perchloric acid in acetonitrile (1 M) to 1.7 mL sample, which was kept in dark for 45 min, was injected in a Shimadzu LC 20 chromatograph (Shimadzu Europa GmbH, Duisburg, Germany). The separation of hydrazones was carried out by using a C18 (NC-04, 250 mm × 4.0 mm ID, 5.0 μm, Prontosil) column (Bischoff Analysentechnik und Geräte GmbH, Leonberg, Germany). The eluent was acetonitrile in water (35%—2 min, 45%—3 min, 100%—20 min and 35%—15 min) at a flow rate of 0.5 mL min^−1^. The retention times and wavelengths used for detection were 18.0 min/350 nm for formaldehyde, 18.8 min/359 nm for acetaldehyde and 19.7 min/360 nm for propionaldehyde. Under the above-mentioned conditions, the derivatization was considered as a quantitative process.

Formaldehyde was also determined spectrophotometrically by using the Hantzsch method. This is based on the reaction among formaldehyde, acetylacetone and ammonium acetate, when the product is a yellow compound that has an absorption maximum at 412 nm of ε(412) = 7700 M^−1^ cm^−1^ [31]. The technique consists of adding 2 mL Hantzsch reagent (25 g ammonium acetate, 3 mL glacial acetic acid and 0.2 mL acetylacetone brought to 100 mL with water) to 10 mL sample, keeping the obtained mixture for 30 min at 50 °C, and measuring the absorbance of the suddenly cooled sample against a blank.

The assessment of hydroxyl radical formation yield with respect to ozone was based on formaldehyde formation following the reaction between *tert*-butanol (acting as a scavenger) and hydroxyl radicals in the presence of oxygen [32]. For the systems where ozone occurs together with oxygen, Flyunt et al. have reported a 50% formaldehyde formation yield with respect to hydroxyl radicals [33].

Given that in our system formaldehyde also resulted following the reaction of 1-propanol with hydroxyl radicals, the calculation of hydroxyl radicals’ formation yield with respect to ozone involves knowing formaldehyde formation yields with respect to ozone in the presence and absence of *tert*-butanol.

Formaldehyde formation yields in the two systems can be derived from the slopes of straight lines in the graphic representation of the formed formaldehyde concentration versus the introduced ozone concentration. An aspect that had to be taken into consideration with this method was the fact that usually isobutene may be present as impurity in *tert*-butanol, which could lead to formaldehyde following the reaction with ozone: (H_3_C)_2_C=CH_2_ + O_3_ + H_2_O → (H_3_C)_2_C=O + H_2_C=O + H_2_O_2_ [34,35]. Given the fact that this reaction was fast, formaldehyde formed at the beginning and thus it did not exert influence on the slopes, but only on the y-intercepts.

Hydrogen peroxide quantification was carried out by using Allen’s method [36]. This was based on the reaction between compounds such as R−O−O−H (R can be an H atom or an organic group) and I^−^, in excess, whereby I_3_^−^ is formed. Given that this is usually a slow reaction, a catalyst, namely ammonium heptamolybdate, is introduced in the system. In the case of H_2_O_2_, the catalyst led to the increase of reaction rate constant from 1.8 × 10^−3^ M^−1^ s^−1^ [33] to 2.1 M^−1^ s^−1^ [34]. Practically, 1 mL Allen’s A (1 g NaOH, 33 g KI and 0.1 g (NH_4_)_6_Mo_7_O_24_·4H_2_O dissolved in Millipore water and brought to 500 mL), 1 mL Allen’s B (10 g potassium hydrogen phthalate dissolved in Millipore water and brought to 500 mL) and 1 mL sample were mixed in a 1 cm cuvette (in between additions the cuvette was strongly stirred). The absorbance of the mixture was read at 350 nm against a blank. The molar absorption coefficient of I_3_^−^ was ε(350) = 25500 M^−1^ cm^−1^ [36].

Quantum chemical calculations were done by using the Density Functional Theory method (DFT) B3LYP [37,38], as implemented in Jaguar program [39]. The structures of the studied molecules were optimized in water with Jaguar’s Poisson-Boltzmann solver (PBF) [40] at B3LYP/6-311+G(d,p)/PBF level of theory. The frequency analysis was carried out to characterize the stationary points on the potential surface and to obtain Gibbs energy (G) at 298 K. The Gibbs energy of reactions (ΔG) were derived from the calculated Gibbs energies of reactants and products. The calculated Gibbs energies give some insight in the feasibility of the reactions. Actually the competition of pathways and product formation are determined by kinetics (for thermodynamically feasible reactions). Calculations of charged species as required for the Gibbs energy of equilibrium are inherently poorer. It was shown [23], that via B3LYP/6–311+G(d,p)/PBF method values comes closest to the experimental ones.

## 5. Conclusions

This study confirms the supposition that ozone reacts with 1-propanol in aqueous media mainly by hydride transfer as expected based on the results for 2-propanol/ozone aqueous system. In contrast to 2-propanol/ozone system, where insertion and H-abstraction have a share of a small percentage, ozone reacts with 1-propanol with yields of one order of magnitude higher as part of the afore-mentioned mechanisms. This is an important finding that linked to the fact that the nature of products depends on mechanism. Thus, the hydride transfer leads to aldehyde and ketone with the same number of carbon atoms as alcohols, while by insertion and H-abstraction, the result is a mix of acids and carbonyl compounds. This means that the same ozone consumption lead to a higher degree of oxidation and to fragmentation of the initial molecule for insertion and H-abstraction (other oxidants such as HO•, HO_2_• and H_2_O_2_ are also formed). In addition, one can notice that the reaction rate between 1-propanol and ozone is four times lower than that between 2-propanol and ozone. The two second order rate constants are: (6.4 ± 0.2) × 10^−1^ M^−1^ s^−1^ for 1-propanol and (2.7 ± 0.1) M^−1^ s^−1^ for 2-propanol. This finding is also correlated with the share of mechanisms.

The comparison between the 2-propanol/ozone and 1-propanol/ozone aqueous systems clearly shows that a small change in the structure of the molecule (in our case switching from a secondary alcohol to a primary one with the same number of carbon atoms) leads to significant variations in the nature and share of the reaction products and also on the reaction rate. This has a great impact, for example, in the treatment of waters. In this context, one may foresee that the water treatment will rely less on the use of some overall parameters and that it will be more and more specific, based on the knowledge of the exact composition of certain waters and the interaction with various chemicals used for water treatment.

## Figures and Tables

**Table 1:** Second order rate constants for the reactions of 1-propanol with various oxidising agents in aqueous media.

Oxidising Agent	K (M^−1^ s^−1^)	Working Conditions	References
**O_3_**	(0.37 ± 0.04)	pH = 2 t = (20 ± 0.5) °C	[1]
	(0.64 ± 0.02)	pH = 7 t = 23 °C	this work
**HO•**	2.8 × 10^9^	pH = 7	[2]
	1.5 × 10^9^	pH = 10.7	[3]
	1.5 × 10^9^	pH = 7	[3]
	2.7 × 10^9^		[4]
**O•^−^**	1.5 × 10^9^	pH = 14	[4]

**Table 2:** Overview of the products formed in 1-propanol/O_3_ aqueous system and their overall formation yields with respect to O_3_.

Product	Propion-Aldehyde	Propionic Acid	Acet-Aldehyde	Acetic Acid	Form-Aldehyde	Formic Acid	Hydroxyl Radical	Hydrogen Peroxyde
Yield (%)	60 ± 3	27.4 ± 1.0	4.9 ± 0.3	0.3 ± 0.1	1.0 ± 0.1	4.6 ± 0.3	9.8 ± 0.3	11.1 ± 0.3

**Table 3:** Second order rate constants for the reactions of O_3_ with various alcohols (literature data).

C–H Bond Type	Alcohol	k_II_(S + O_3_)	
		Value (M^−1^ s^−1^)	Reference
RH_2_C−H	(H_3_C)_2_(HO)C H_2_C−H (*tert*-Butanol)	1.1 × 10^−3^	[6] *
		3 × 10^−3^	[1] **
	(HO)H_2_C−H (Methanol)	2.4 × 10^−2^	[1] **
R(HO)HC−H	(H_3_C)(HO)HC−H (Ethanol)	(3.7±0.4) × 10^−1^	[1] **
	(H_5_C_2_)(HO)HC−H (1-Propanol)	(6.4 ± 0.2) × 10^−1^	this work * [1] **
		(3.7 ± 0.4) × 10^−1^	
	(H_7_C_3_)(HO)HC−H (1-Butanol)	(5.8 ± 0.6) × 10^−1^	[1] **
	(H_15_C_7_)(HO)HC−H (1-Octanol)	˂8 × 10^−1^	[1] **
R_2_(HO)C−H	(H_3_C)_2_(HO)C−H (2-Propanol)	(2.7 ± 0.1)	[5] * [1] **
		(1.9 ± 0.2)	
	(H_2_C)_4_(HO)C−H (Cyclopentanol)	(2 ± 0.2)	[1] **

*—measured at 23 °C; **—measured at 20 °C.

## Supplementary Materials

Supplementary materials can be found at https://www.mdpi.com/1422-0067/20/17/4165/s1.
