# Supplementary material for: Reaction of 1-propanol with Ozone in Aqueous Media
Source: Int J Mol Sci. 2019 Aug 26;20(17):4165. doi: 10.3390/ijms20174165 (PMC6747496; doi:10.3390/ijms20174165)
Supplement: Supplementary file 1 [file ijms-20-04165-s001.pdf]

International Journal of
Molecular Sciences

MDPI

Experimental

All chemicals were p.a. grade from: Merck (Merck Millipore, Darmstadt, Germany), Fluka (Fluka Chemie GmbH, Buchs, Switzerland), Fischer (Fischer Scientific GmbH, Schwerte, Germany), Alfa Aesar (Alfa Aesar, Karlsruhe, Germany) or Sigma Aldrich (Sigma Aldrich Chemie GmbH, München, Germany) and they were used without further purification.

Table 1. Reagents and their sources.

Method	Reagents	Source
Formaldehyde determination by using the Hantzsch method	Formaldehyde 37%	Merck Millipore, Darmstadt, Germany
	Acetylacetone	Merck Millipore, Darmstadt, Germany
	Ammonium acetate	Merck Millipore, Darmstadt, Germany
	Glacial acetic acid	Merck Millipore, Darmstadt, Germany
Formaldehyde, acetaldehyde and propionaldehyde determination by using DNPH method (HPLC)	Formaldehyde 37%	Merck Millipore, Darmstadt, Germany
	Acetaldehyde	Fluka Chemie GmbH, Buchs, Switzerland
	Propionaldehyde	Merck Millipore, Darmstadt, Germany
	2,4-dinitrophenylhydrazine (2,4-DNPH)	Merck Millipore, Darmstadt, Germany
	Acetonitrile	Fischer Scientific GmbH, Schwerte, Germany
	Perchloric acid 70%	Sigma Aldrich Chemie GmbH, München, Germany
Formate, acetate and propionate determination (IC)	Sodium formate	Alfa Aesar, Karlsruhe, Germany
	Sodium acetate	Alfa Aesar, Karlsruhe, Germany
	Sodium propionate	Merck Millipore, Darmstadt, Germany
	Sulphuric acid 98%	Merck Millipore, Darmstadt, Germany
Hydrogen peroxide determination by means of Allen's method	Hydrogen peroxide 30%	Merck Millipore, Darmstadt, Germany
	Sodium hydroxide	Merck Millipore, Darmstadt, Germany
	Potassium iodide	Merck Millipore, Darmstadt, Germany
	Ammonium heptamolybdate	Merck Millipore, Darmstadt, Germany
	Potassium hydrogen phthalate	Merck Millipore, Darmstadt, Germany
General purpose	1-Propanol	Alfa Aesar, Karlsruhe, Germany
	tert-Butanol	Merck Millipore, Darmstadt, Germany

Reaction of ozone with HO• in the 1-propanol / ozone system

Taking into account that the second order rate constant for the reaction of HO• with O_3 was more than one order of magnitude lower than that of HO• with 1-propanol {k(HO• + O_3) = 1.1 × 10^8 M^{-1} s^{-1} [1] and k(HO• + 1-propanol) = 2.7 × 10^9 M^{-1} s^{-1} [1, 2]} and that in all experiments the ratio between 1-propanol and ozone concentrations was higher than 10, it followed that the ratio between the pseudo first order rate constants (observed rate constants) for the reaction of ozone with HO• and that for the reaction of 1-propanol with HO• was less than 1:100:

$$\frac{k_{obs.}(O_3 + HO\cdot)}{k_{obs.}(1 - propanol + HO\cdot)} = \frac{k_{II}(O_3 + HO\cdot) \times [O_3]}{k_{II}(1 - propanol + HO\cdot) \times [1 - \text{propanol}]} <$$

$$\frac{1.1 \times 10^8\ \text{M}^{-1}\text{s}^{-1} \times [O_3]}{2.7 \times 10^9\ \text{M}^{-1}\text{s}^{-1} \times 10 \times [O_3]} < \frac{1}{100}$$

This showed clearly that the ozone decay via HO• was negligible. This finding is also illustrated in Figure 1 where the decay of ozone in water without any reagent (curve 2) and in the systems 1-propanol / ozone (curve 1) and *tert*-butanol / ozone (curve 3) are depicted. The pseudo first order rate constant for the reaction between 1-propanol and HO• {k_{obs}(HO• + 1-propanol) = 2.7 × 10^9 M^{-1} s^{-1} × 36 × 10^{-3} M = 9.7 × 10^7 s^{-1}} was higher than that for the reaction between *tert*-butanol and HO• {k_{obs}(HO• + *tert*-butanol = 6 × 10^8 M^{-1} s^{-1} × 20 × 10^{-3} M = 1.2 × 10^7 s^{-1}}, which meant that HO• scavenging effect in 1-propanol / ozone – system was more effective than in *tert*-butanol / ozone system. In other words, ozone decomposition curve following the reaction with HO• in 1-propanol / ozone system was above curve 3 (corresponding to ozone decomposition following its reaction with HO• in *tert*-butanol / ozone system; in the latter system the direct reaction between ozone and the substrate was negligible). As a consequence, ozone decay in 1-propanol / ozone system, depicted by curve 1, was virtually due to the direct reaction between ozone and 1-propanol.

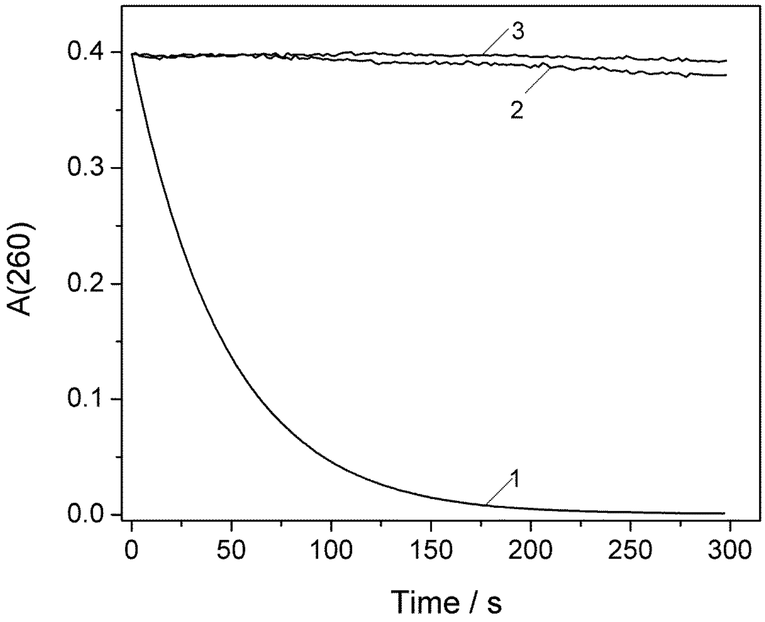

Figure 1. Decrease of ozone concentration vs. time measured at 260 nm absorbance for various aqueous systems: 1 - 1-propanol / ozone, with [1-propanol] = 36 mM; 2 - only ozone; 3 - *tert*-butanol / ozone, with [*tert*-butanol] = 20 mM.

Decay of α-hydroxyalkylperoxyl radical by $HO_2\bullet$ / $O2\bullet^-$ elimination

Bothe et al. have described α-hydroxyalkylperoxyl radical decay via a transition state that contains a five membered ring (reactions (1) and (2)) [3, 4].

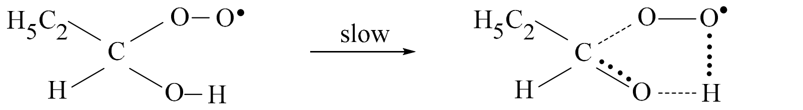

(1)

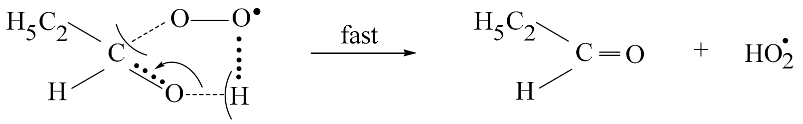

(2)

Depending on pH, the formed $HO_2\bullet$ deprotonates according to equilibrium (3) that is characterised by pKa = (4.8±0.1) [5]. This means that by this mechanism, even at higher pH, α-hydroxyalkylperoxyl radical (not the corresponding radical anion) is the species that decays and that $O_2\bullet^-$ forms only at the end, by deprotonation of the previously eliminated $HO_2\bullet$.

$$HO_2\bullet \rightleftarrows O_2\bullet^- + H^+ \qquad (3)$$

Given that a cyclic transition state is involved in this mechanism, it explains the experimental finding that the rate constant does not depend on solvent.

According to another theory at higher pH values (what "higher" means depends on pKa value of α-hydroxyalkylperoxyl radical i.e. on the nature of substituents), the α-hydroxyalkylperoxyl radical deprotonates according to equilibrium (4) and then the deprotonated species undergoes $O_2\bullet^-$ elimination, as in reaction (5) [6, 7].

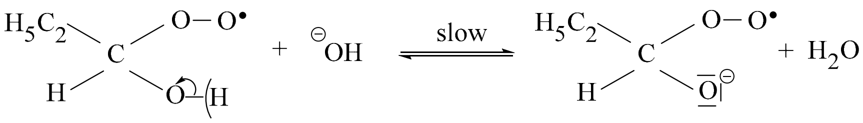

(4)

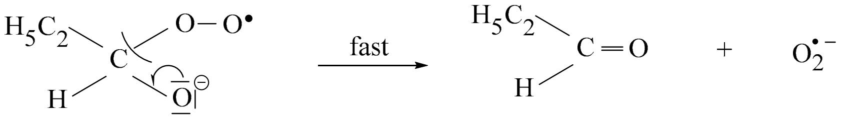

(5)

In the above mentioned succession of reactions, the first step is the rate limiting one and that means that the reaction rate of the overall process, *r*, is practically equal with that of deprotonation, r_{4+}:

$$r = r_{4+} = \frac{d[R_1R_2C = O]}{dt} = k_{4+} \cdot [HO^-] \cdot [R_1R_2(HO)COO \cdot]$$

It follows that $O_2\bullet^-$ elimination from α-hydroxyalkylperoxyl radical anion occurs according to a second order kinetics, which is in line with the literature [6, 7].

Bimolecular decay of α-hydroxyalkylperoxyl radicals. Russel and Bennett mechanisms

Besides $HO_2\bullet$ elimination, primary and secondary α-hydroxyalkylperoxyl radicals can undergo bimolecular decay that involves the formation of a short lived intermediate - a tetroxide (I). This process occurs via a first transition state (TS_1). The tetroxide further can eliminate O_2, via two pathways, or H_2O_2.

Oxygen elimination can lead to two α-hydroxyalkyloxyl radicals (first pathway) or to propionaldehyde and propionic acid in a ratio of 1:1 (second pathway). The latter is known in the literature as Russel reaction and it involves a transition state (a second one; TS_2) where the four oxygen atoms of tetroxide, a carbon and a hydrogen atom form a six membered ring [8]. Given that this ring includes one hydrogen atom, only primary and secondary α-hydroxyalkylperoxyl radicals can undergo this kind of oxygen elimination.

The transformation of reagents into products according to the Russel mechanism is shown in the following succession of reactions.

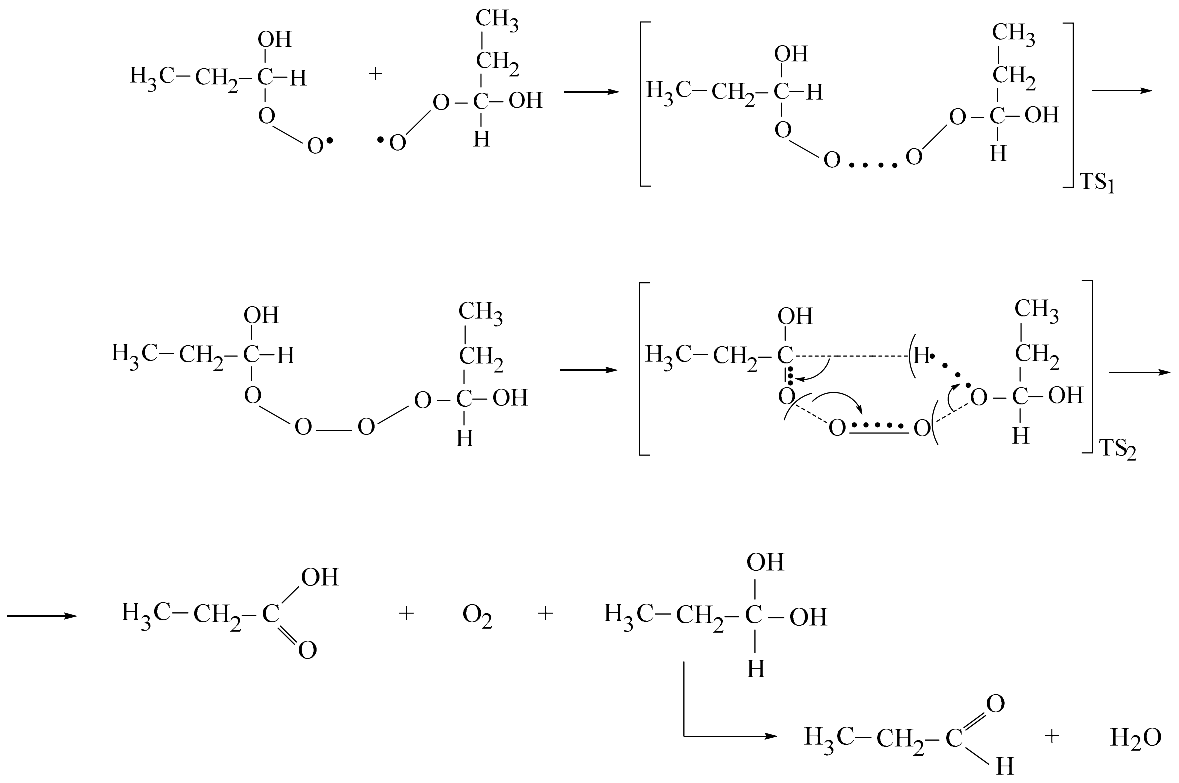

Hydrogen peroxide elimination has been explained by Bennett and that is why this reaction has been named accordingly. Bennett reaction implies the formation of a transition state that contains two five-membered rings, each one consisting of three oxygen atoms, one carbon and one hydrogen atom; two of the oxygen atoms are shared between the rings [7, 9, 10]. One can note that also in this case each ring contains one hydrogen atom, which will be included in the eliminated hydrogen peroxide and it follows that this reaction also occurs only in the case of primary and secondary α-hydroxyalkylperoxyl radicals.

The way α-hydroxyalkylperoxyl radical decays via Bennett mechanism is shown below. This succession highlights that this mechanism also implies an intermediate (tetroxide) and two transition states and from this point of view it follows the same pattern as the Russel mechanism.

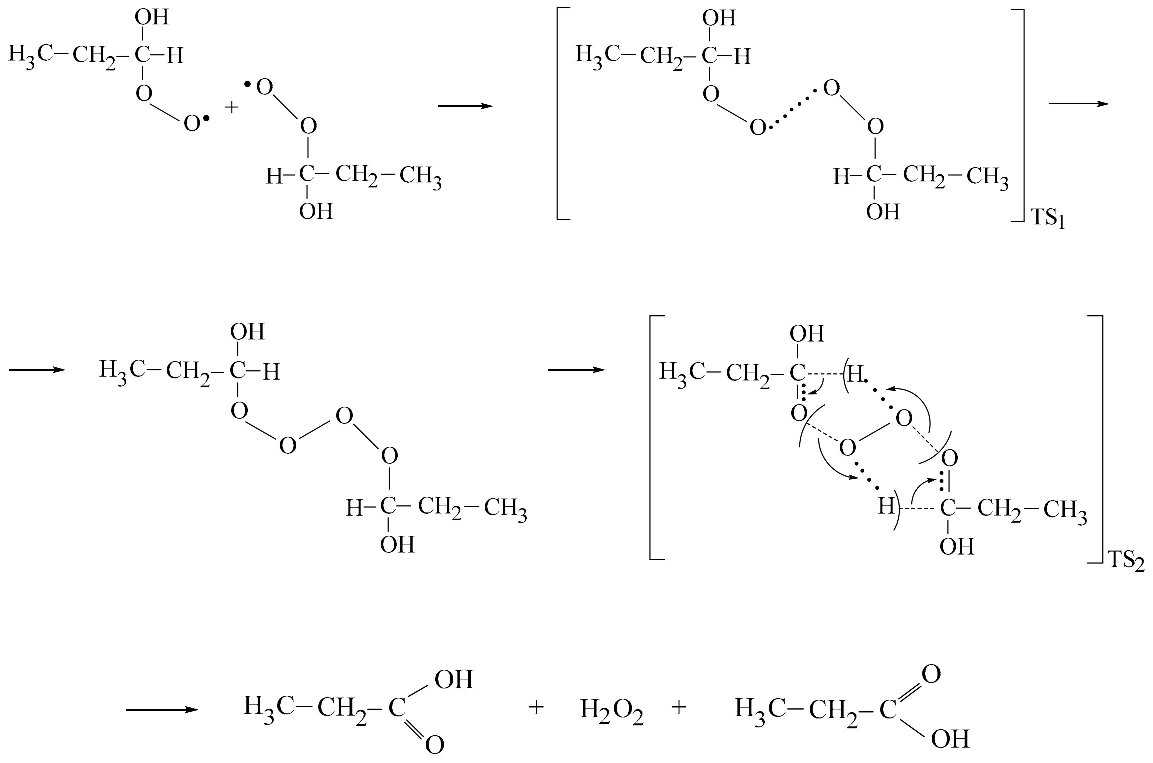

As already shown, both Russel and Bennett mechanisms involve the formation of an intermediate (I) and two transitions states (TS_1 and TS_2):

$$2\ R \rightarrow [TS_1] \rightarrow I \rightarrow [TS_2] \rightarrow Products$$

The energy diagram for the decay of α-hydroxypropylperoxyl radical according to Russel and Bennett mechanisms is given in Figure 2.

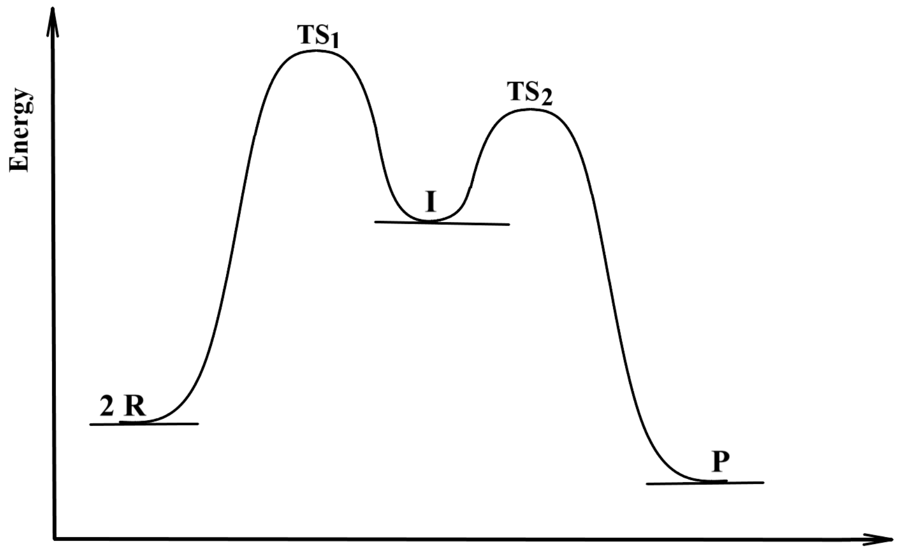

Figure 2. Schematic representation of the energy diagram for the decay of α-hydroxypropylperoxyl radical (Russel and Bennett mechanisms). R stands for reactants, TS_1 and TS_2 for transition states, I for intermediate and P for products.

References

1. Buxton, G.V.; Greenstock, C.L.; Helman, W.P.; Ross, A.B. Critical Review of Rate Constants for Reactions of Hydrated Electrons, Hydrogen atoms and Hydroxyl Radicals ($HO\bullet/O\bullet^-$) in Aqueous Solution. *J. Phys. Chem. Ref. Data* **1988**, *17*, 513-886.
2. Neta, P.; Schuler, R.H. Rate Constants for the Reaction of $O\bullet^-$ Radicals with Organic Substrates in Aqueous Solution. *J. Phys. Chem*. **1975**, *79*, 1-6.
3. Bothe, E.; Behrens, G.; Schulte-Frohlinde, D. Mechanism of the First Order Decay of 2-Hydroxy-propyl-2-peroxyl Radicals and of $O_2\bullet^-$ Formation in Aqueous Solution. *Z. Naturforsch*. **1977**, *32 b*, 886-889.
4. Bothe, E.; Schulte-Frohlinde, D.; von Sonntag, C.. Radiation Chemistry of Carbohydrates. Part 16. Kinetics of $HO_2\bullet$ Elimination from Peroxyl Radicals derived from Glucose and Polyhydric Alcohols. *J. Chem. Soc., Perkin Trans.* 2. **1978**, 416–420.
5. Bielski, B.H.J.; Cabelli, D.E.; Arudi, R.L.; Ross, A.B. Reactivity of HO_2/O_2^- Radicals in Aqueous Solution. *J. Phys. Chem. Ref. Data* **1985**, *14*, 1041-1100.
6. von Sonntag, C.; Schuchmann, H.-P. Peroxyl Radicals in Aqueous Solutions. In: *Peroxyl Radicals*, Z. B. Alfassi (ed.); John Wiley & Sons Ltd.: Chichester, **1997**, 173-234.
7. von Sonntag, C.; von Gunten, U. *Chemistry of Ozone in Water and Wastewater Treatment: From Basic Principles to Applications*, International Water Association Publishing: London, U.K., **2012**.
8. Russel, G.A. Deuterium-isotope Effects in the Autoxidation of Aralkyl Hydrocarbons. Mechanism of the Interaction of Peroxyl Radicals. *J. Am. Chem. Soc.* **1957**, *79*, 3871-3877.
9. Bennett, J.E.; Summers, R. Product Studies of the Mutual Termination Reactions of sec-Alkylperoxy Radicals: Evidence for Non-cyclic Termination. *Can. J. Chem*. **1974**, 52, 1377-1379.
10. Bothe, E.; Schulte-Frohlinde D. The Bimolecular Decay of the α-Hydroxymethylperoxyl Radicals in Aqueous Solution. *Z. Naturforsch.* **1978**, *33 b*, 786-788.
